# An inexpensive and rapid diagnostic method for detection of SARS-CoV-2 RNA by loop-mediated isothermal amplification (LAMP)

**DOI:** 10.1016/j.mex.2023.102011

**Published:** 2023-01-11

**Authors:** S Prakash, Priyatma, R Aasarey, P K Pandey, P Mathur, S Arulselvi

**Affiliations:** Department of Laboratory Medicine, All India Institute of Medical Sciences, New Delhi, 110029, India

**Keywords:** SARS-CoV-2, LAMP, Covid-19, Blood, CBC, Colorimetric assay, NP, OP, Loop Mediated Isothermal Amplification (LAMP)

## Abstract

SARS-CoV-2 is a public pandemic health concern globally. Nasopharyngeal and oropharyngeal swab samples are used for Covid-19 viral detection. Sample collection procedure was tedious and uncomfortable and unsuitable for biochemical and CBC analysis in swab samples. Biochemistry and CBC tests are key determinant in management of Covid-19 patients. We developed a LAMP test to detect viral RNA in blood samples. LAMP is required four specific primers targeting the internal transcribed S-region and loop primers for viral RNA amplification. RNA was extracted from blood samples by TRIzol method. LAMP reaction was performed at 60 °C for 1 hour and amplicons were visualized in HNB dye. No cross-reactivity was seen with HBV, HCV, and HIV infected sample. Out of 40 blood samples, 33 samples were positive for LAMP and Q-PCR analysis, one sample was positive for LAMP and negative for Q-PCR, two samples were negative for LAMP but positive for Q-PCR, and four blood samples were negative for LAMP and Q-PCR. LAMP method has an accuracy of 92.50%, with sensitivity and specificity of 94.28% and 80%, respectively. Thus, LAMP diagnostic test has proved reliable, fast, inexpensive and can be useful for detection where the limited resources available.

• LAMP method is a potential tool for detection of SARS-CoV-2.
• Blood samples are the key determinant for routine diagnostics as well as molecular diagnostics.
• LAMP assay is an appropriate diagnostics method which offers greater simplicity, low cost, sensitivity, and specificity than other methods in molecular diagnostics.

## Graphical abstract

**Figure:**
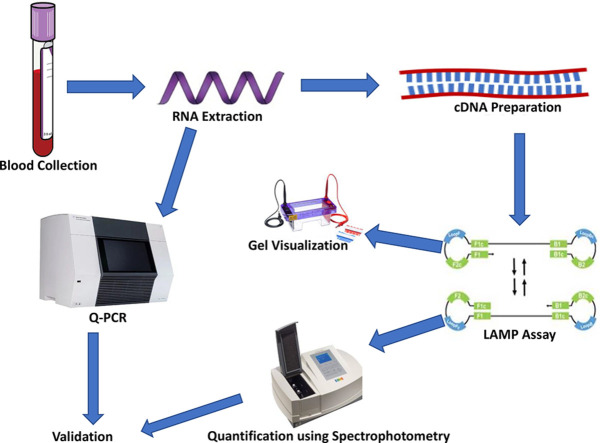

Specification Table

**Table:** 

Subject Area:	Biochemistry, Genetics and Molecular Biology
Specific Subject Area:	Molecular Biology
Method Name:	Loop Mediated Isothermal Amplification (LAMP)
Name and Reference of Original Method:	• Loop Mediated Isothermal Amplification (LAMP) • T. Notomi, H. Okayama, H. Masubuchi, T. Yonekawa, K. Watanabe, N. Amino, T. Hase, Loop-mediated isothermal amplification of DNA., Nucleic Acids Res. 28 (2000) E63. https://doi.org/10.1093/nar/28.12.e63.
Resource Availability:	• The Funding for this project was received from Intramural Grant of AIIMS, New Delhi, India • (A-COVID-9). • Institutional ethical clearance was obtained from the ethics committee (IEC-289/17.04.2020). • Blood samples of patients were collected for routine chemistry analysis and LAMP study from COVID Ward of JPN Trauma center, AIIMS, New Delhi, India. • All experiments and tests were performed in well-established labs of AIIMS New Delhi.

## Introduction

The emergence of viral diseases poses a severe threat to public health globally. Several viral epidemics have frequently occurred in recent decades, including severe acute respiratory syndrome coronavirus (SARS-CoV), H1N1 influenza, and coronavirus with the Middle East Respiratory Syndrome (MERS-CoV) [1]. Severe Acute Respiratory Syndrome Coronavirus 2 (SARS-CoV-2) has rapidly evolved into a global public health emergency since its outbreak in Wuhan, China, in December 2019. SARS-CoV-2 is an enveloped, unsegmented, positive-sense single-stranded RNA virus. The virus is highly transmitted from human to human, while primarily transmission is found through droplets by coughing and sneezing. Speaking or breathing may be enough source to transmit the SARS-CoV-2 virus [2].

However, early diagnosis of SARS-CoV-2 is essential to control disease progression and limit the spread of the virus in the community. It is used as part of a mitigation strategy to stop future infections and reduce the burden on the healthcare system [3]. The gold standard for the COVID-19 test is reverse transcriptase quantitative- PCR (Q-PCR), which detects the genetic material for SARS-CoV-2 from the nasopharyngeal (NP) and oropharynx (OP) swab samples. Q-PCR diagnostics are reliable but complex, expensive, and time-consuming methods. In addition, due to its global expansion, there was a shortage of reagents and consumables required for processing samples to detect viral RNA in the early stages of a pandemic [4]. Since good quality and adequate amount of RNA are required to perform the viral detection levels in Q-PCR, PCR, and other available methods, they are time-consuming. They need well-furnished infrastructure, highly skilled technical staff, costlier reagents, kits, etc.

**Fig. 1:**
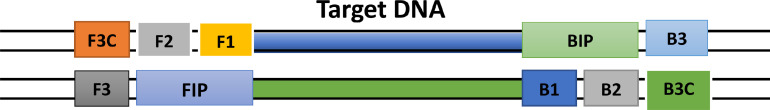
Primer Binding sites: F3 and B3 are outer primers that bind to F3C and B3C regions. Forward Inner primers (FIP) binds to F1 and F2 regions, and backward inner primer (BIP) binds to the B1 and B2 regions of the template.

These limitations are exacerbated by the rapid expansion of the pandemic, as Q-PCR does not have the screening capacity to catch up. Many nations have developed protocols such as containment and mitigation activities to protect against viral spread within the population to stop onward transmission and decrease the load on the healthcare system. There is a need to find an alternative method that is rapid, accurate, cost-effective, and less time-consuming for quantifying viral load. Therefore, a new solution for COVID-19 detection is in high demand, and one way seems to be the loop-mediated isothermal amplification (LAMP) method. Due to its simplicity, specificity, rapidity, and low cost, it is a well-established technology in various fields such as medicine, agriculture, and the food industry. LAMP is a nucleic acid amplifier under isothermal conditions, highly compatible with point-of-care (POC) analysis, and can improve diagnosis [5]. Notomi and his colleagues developed the loop-mediated isothermal amplification (LAMP) method to amplify the specific regions of targeted viral DNA under isothermal conditions in 2000 [6]. The concept of the method was to provide specific, sensitive, and rapid detection of DNA/RNA in the diagnostic field. Since PCR/RT-PCR was the only advisable method for detecting the DNA/RNA, that requires highly skilled technical, well-developed infrastructure, and time taking method; therefore, due to cautions of pandemic LAMP method can be a rapid alternative to Q-PCR methods. That requires four primers, i.e., FIP (forward Inner Primer), BIP (Backward Inner Primer), F3 (Forward Primer), and B3 (Backward Primer), *Bst* enzyme, and bind to six different template strand regions, as shown in Fig. 1. However, to increase the stability and amplification rate of the reaction, two additional primers are used, i.e., LF (forward Loop) and LB (Backward Loop) [7].

Since the reaction releases magnesium pyrophosphate (reaction by-product) in proportion to the amount of amplification product, with the interaction of hydroxy naphthol blue dye and endpoint detection can be performed with the naked eye, especially when the reaction amount is significant precipitated and turned from the violet color to sky blue color. Depending on the intensity of color, the positivity rate of the viral load qualitative can be assessed. Simultaneously the precipitated complex color reaction mixture can be measured spectrophotometrically or ELISA reader at 648 nm.

As saliva and NP samples are potential biological samples for quantifying the COVID-19 virus, their sample collection procedure was uncomfortable for the patient. Based on the above background and tedious process for sample collection, this study was planned to assess the viral load in blood samples. It will help to quantify viral load as well as other biochemical and hematological parameters, that have an essential role in managing severity in Covid-19 infection. Thus, the LAMP method is an alternative to Q-PCR that is faster, rapid, cost-effective, and less time-consuming for qualitative and quantitative viral load in the pandemic situations.

## Materials and methodology

(A) **Sample Collection-** All admitted patients' blood samples were collected at JPN Trauma Center, AIIMS, New Delhi, that designated only for SARS-CoV-2 infected patients. Blood samples were collected for routine biochemical, cytokines, and CBC analysis to assess the severity of SARS-CoV-2 viral status. Patients who recently encountered the COVID-19 viral infection were recruited as test samples, and healthy people's blood was chosen as control subjects. The blood samples collected from both test and control patients in EDTA and plain vials for routine blood chemistry, hematology parameters, cytokines, and C-reactive protein (CRP). The liver function and serological profile were also done in an automated chemistry analyzer (ROCHE diagnostics). Institutional ethical clearance was obtained from the ethics committee (IEC-289/17.04.2020).
(B) **Sample Size Calculation-** The sample size was calculated from the Department of Biostatistics, AIIMS, New Delhi, and the sample size was calculated at 40 numbers.
(C) **RNA Isolation -** RNA is isolated from whole blood samples using the TRIzol Isolation method [8]. TRIzol, a homogenous solution of phenol and guanidium isothiocyanate, was used to solubilize the biological materials and denature the protein. Chloroform was used for phase separation, i.e., aqueous phase for RNA, organic phase for protein, and nucleic acids at the interphase layer. Subsequent steps were followed, and finally collected RNA, and their quality was checked from a nanodrop spectrophotometer.
(D) **Primer Design-** A total of six primers, including both external primers (F3 and B3), inner primer (FIP and BIP), and loop primers (LF and LB), were designed using LAMP designer software Premier Bio-soft, USA, which recognizes a total of eight different regions on S gene of SARS-CoV-2 template. These primers were synthesized by IDT (Integrating DNA Technology), Canada. The Primer sequence is shown in Table 1.

**Table 1:** Primer sequence of S-gene region of SARS-CoV-2 RNA.

**LAMP Primers Sequence (5´−3′)**	
F3	TGG TGA TAT TGC TGC TAG A
B3	GCA CTA TTA AAT TGG TGG GC
FIP	AGG TCC AAC CAG AAG TGA TTC ACC TTT GCT CAC AGA TG
BIP	GCA GGT GCT GCA TTA CAA TCT GTG TAA CTC CAA TAC CA
LF	GCT AAC AGT GCA GAA GTG TAT T
LB	GCT ATG CAA ATG GCT TAT AGG T
**Q-PCR Primers Sequence (5´−3´)**	
F	TGG TGA TTG CCT TGG TGA TAT TGG
R	CCA GAA GTG ATT GTA CCC GCT AAC
(E) **cDNA Preparation-** cDNA is double-stranded DNA synthesized using single-stranded RNA as a template strand using the reverse transcriptase enzyme. The reaction mixture was prepared using the RNA template, reverse transcriptase enzyme, random Primers, dNTPs, RT buffer, and RNase-free water and placed in a digital water bath at 45 °C for 20 min for the reverse transcription steps.
(F) **LAMP Assay-** The LAMP assay amplifies a gene in the form of dsDNA. The reaction process for gene amplification and detection is performed in a single step by incubating a mixture of primers, dNTPs, HNB (Hydroxy Naphthol Blue), Betaine, MgSO_4_, *Bst* DNA polymerase with strand displacement or replacement activity, and substrate at a constant temperature (approximately 65 °C) for 50–60 min (Fig. 2).

**Fig. 2:**
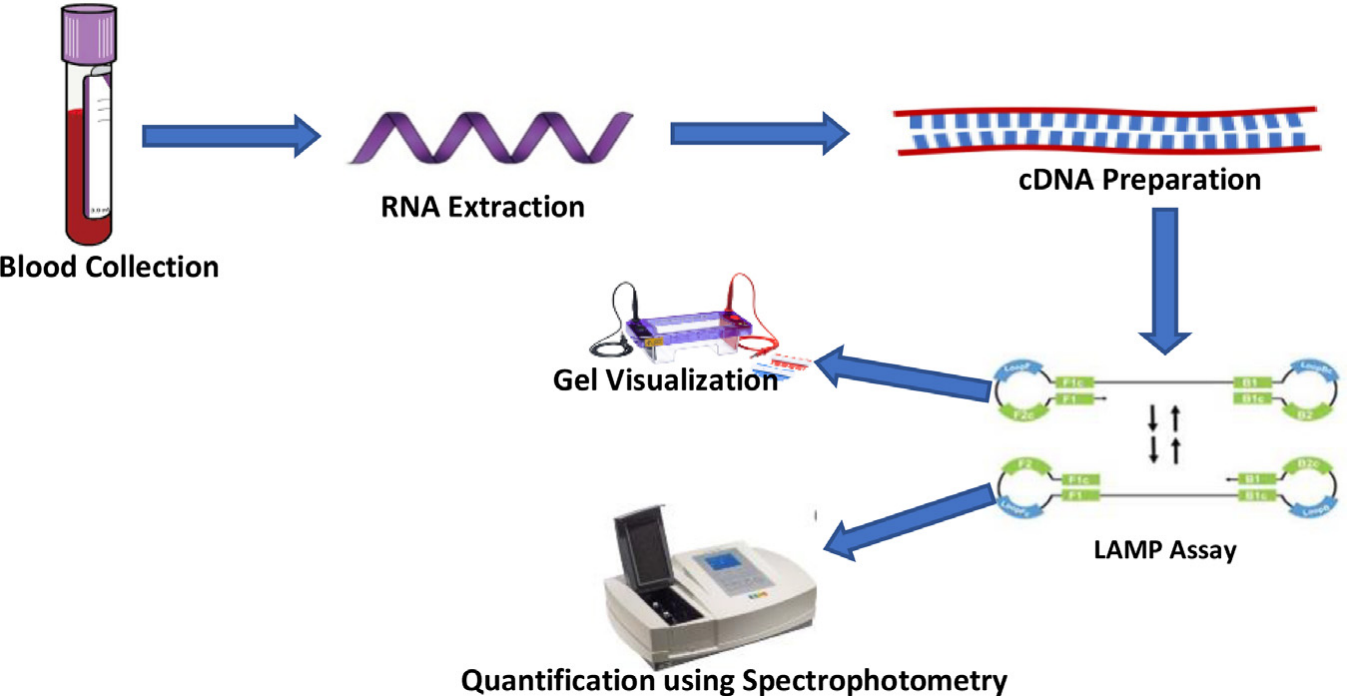
Schematic representation of working protocol.

To brief on LAMP amplification, it begins when the F2 region of FIP hybridizes to the F2c region of the target DNA template and initiates complementary strand synthesis, followed by the F3 primer hybridizing to the F3c region of the target DNA for elongation and displacing the FIP-linked complementary strand. This displaced strand forms a loop at the 5′end. This single-stranded DNA with a loop at the 5′end serves as a template for BIP. B2 hybridizes to the B2c region of the template DNA. DNA synthesis is initiated, complementary strands are formed, and the 5′end loop opens. Subsequently, B3 hybridizes to and extends to the B3c region of the target DNA, displacing the complementary strand bound to BIP [9]. This creates dumbbell-shaped DNA. Nucleotides are added to the 3′end of F1 by the *Bst* DNA polymerase, which is also responsible for expanding and opening the Loop at the 5′end. Dumbbell-shaped DNA is converted to a stem-loop structure. This structure acts as an initiator for the LAMP cycle, the second stage of the LAMP reaction. Loop primers were also added for exponential amplification of LAMP. The final product obtained is a mixture of stem-loop DNA with different stem lengths and different cauliflower-like multi-loop structures.

The reaction is much faster and releases magnesium pyrophosphate (reaction by-product) in proportion to the amount of amplified production in amalgamation with HNB depending upon the viral load, allowing the endpoint to be seen with the naked eye and quantitatively assess the concentration of viral load at 648 nm by ELISA reader or spectrophotometers against no template.

### Statistical analysis

Diagnostic studies were analyzed based on the extracted true positive (TP), false positive (FP), false negative (FN), and true negative (TN) data. Diagnostic performance of Q-PCR and LAMP uses summary accuracy, sensitivity (Se), specificity (Sp), likelihood ratio (LR), and prevalence were evaluated [10].

The equations for calculations are as follows:

**Accuracy =** (TP+TN)/n

**Sensitivity =** TP/ (TP+FN)

**Specificity =** TN/ (TN+FP)

**Prevalence of Disease =** T_Disease_/n

**Positive Likelihood Ratio =** Sensitivity/ (100-Specificity)

**Negative Likelihood Ratio =** (100-Sensitivity)/Specificity

Where ***n***= Total no. of samples, **TP**= True Positive, **TN**= True Negative, **FN**= False Negative, and **FP**= False Positive.

## Result

### Screening of patients

The complete blood count was done in all patients. In patients with SARS-CoV-2 Q-PCR positive, the blood parameters were analyzed, such as hemoglobin (Hb), hematocrit (HCT), alanine aminotransferase (ALT), and aspartate transferase (AST), C-reactive protein (CRP), ferritin and interleukin-6 (IL-6) as shown in Table 2.

**Table 2:** Hematological parameters of patients and healthy control.

Group	Hb (g/dL)	HCT (%)	ALT (U/L)	AST (U/L)	CRP (mg/L)	Ferritin (ng/mL)	IL-6 (pg/mL)
**Patients** (*n* = 40)	10.2 ± 2.61	31.48 ± 8.10	48.5 ± 49.62	54.41 ± 48.51	4.95 ± 5.58	520.28 ± 366.48	95.68 ± 133.31
**Healthy Control** (*n* = 25)	12.8 ± 2.46	33.28 ± 2.65	26.2 ± 8.42	22.4 ± 6.82	0.4 ± 0.12	65.8 ± 32.2	8.98 ± 3.65

**Fig. 3:**
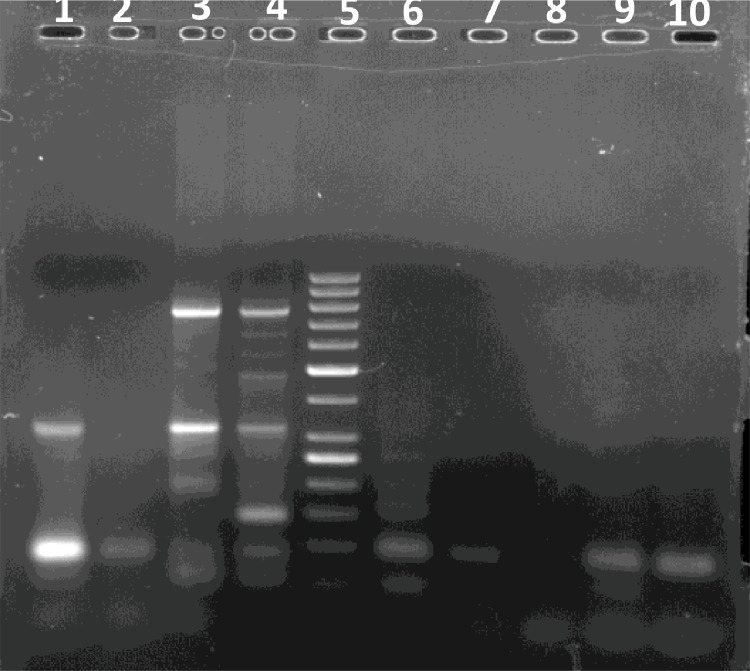
PCR product of COVID-19, Lane1–4 & 6 PCR product at the combination of primers, Lane 5- DNA Ladder, Lane 8- NTC, and Lane 9–10 positive samples.

Before performing the LAMP method, the designed and synthesized primers were checked in PCR for the primer activity. Q-PCR reaction was performed using master mix containing PCR buffer (1X, 2.5 µl), MgCl_2_ (1 mM, 1 µl), dNTPs Mix (10 mM, 0.5 µl), forward primer (1 mM, 0.25 µl), reverse primer (1 mM, 0.25 µl), Taq polymerase (1 U/Reaction, 0.2 µl) and nuclease-free water (15.3 µl). The thermal profile for PCR reaction was kept as hot start (95 °C/3 min), tap (95 °C/3 min), short cycle (40 Times), denaturation (95 °C/30 s), annealing (58 °C/30 s), elongation (72 °C/30 s) with end temperature (72 °C/7 min). The amplified products were visualized by 1% agarose gel in the gel documentation system, as shown in Fig. 3.

**Fig. 4:**
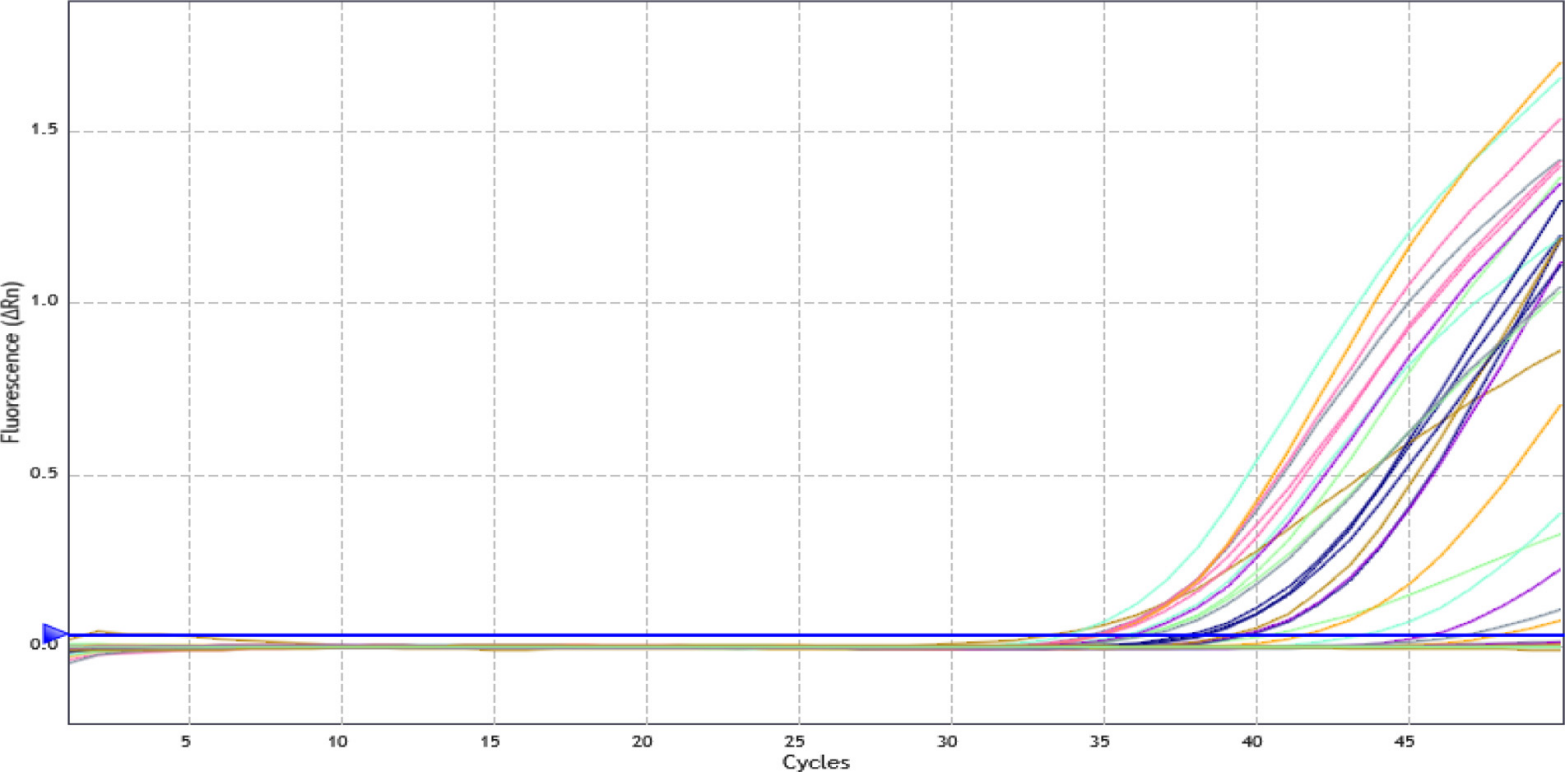
Amplification plot of SARS-CoV-2 samples.

Similarly, viral load quantification was done using SYBR mix reaction buffer, primers (F&R), and template. The thermal condition of the reaction was the hot start (95 °C/3 min), tap (95 °C/3 min), short cycle (40 Times), denaturation (95 °^C^/30 s), annealing (58 °C/30 s), elongation (72 °C/30 s) with end temperature (72 °C/7 min). These samples were amplified, and their amplification and melting curves confirm the presence of viral load (shown in Fig. 4). The Ct values were ranged from 33 to 38 in Covid-19 infected samples and rest may have low copy no. of viral RNA.

**Fig. 5:**
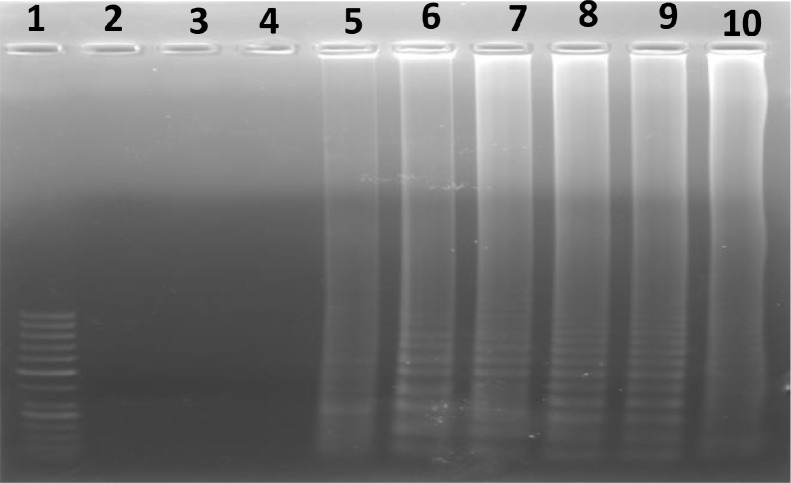
Gel Electrophoresis of samples, amplified in LAMP method. Well 1 contains DNA ladder, well2 contains Non-Template Control, well3 has Healthy control sample, well4 contains disease control samples, and well 5–10 contains covid-19 positive samples.

The same samples were performed for the LAMP reaction. The reaction mixture solution for LAMP was prepared using master mix (1X, 10 µl), MgSO_4_ (1 mM, 0.8 µl), tween-20 (0.005%, 0.10 µl), dNTP (1.25 mM, 2.5 µl), betaine (100 mM, 0.40 µl), forward primer (F, 0.60 µl), forward internal primer (FIP, 1.20 µl), backward primer (B, 0.60 µl), backward internal primer (BIP, 1.20 µl), loop forward primer (LF, 0.40 µl), loop backward primer (LB, 0.40 µl), *Bst* polymerase (8 U/Reaction, 1 µl), and cDNA (0.8 µl). The mix was spun and incubated at 60 °C for 1 hour. The samples were then run in Gel (1% Agarose) electrophoresis to get the gene amplification. The image of Gel is shown below in Fig. 5.

In the LAMP reaction, the accumulation of magnesium pyrophosphate (a by-product) has resulted in the white turbidity of the reaction mixture. The results of LAMP amplification were visually detected with the addition of 120 µM HNB dye.

(DNA)_n-1_+dNTP → (DNA)_n_ + P_2_O_7_^4−^

P_2_O_7_^4−^ + 2Mg^2+^ → Mg_2_P_2_O_7_ ↓ **White PPT**

Mg_2_P_2_O_7_ + HNB (Violet color) → Sky blue

**Fig. 6:**
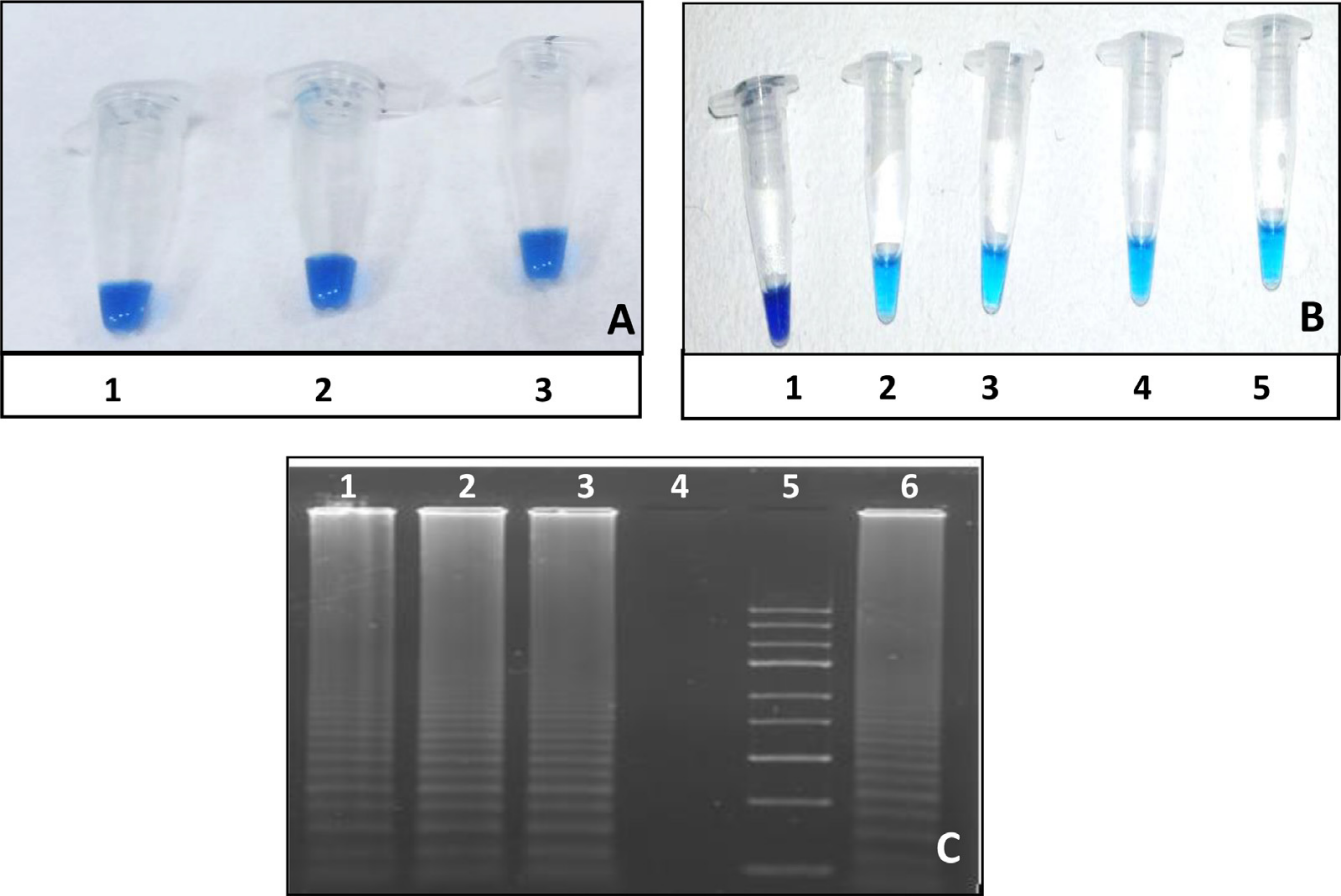
Amplification images of LAMP products.

HNB dye reacts with the free Mg^2+^ ions and produces a violet color. As the amplification proceeds, free Mg^2+^ ion converts into magnesium pyrophosphate with the interaction of hydroxy naphthol blue dye turns to light sky blue as shown in Fig. 6B; on the other hand, the amplification of the same samples was visualized in 1% agarose gel in gel documentation system as shown in Fig. 6C.

• LAMP reaction mixture with HNB before the reaction proceedings
• LAMP reaction mixture with HNB after the reaction process, tube 1 consists of NTC and other tubes of Positive covid-19 samples.
• Image of the amplified HNB product. Well, 1, 2, 3 & 6 consists of COVID-19 positive samples, four consists of non-template controls, and well 5 contain a DNA ladder.

**Fig. 7:**
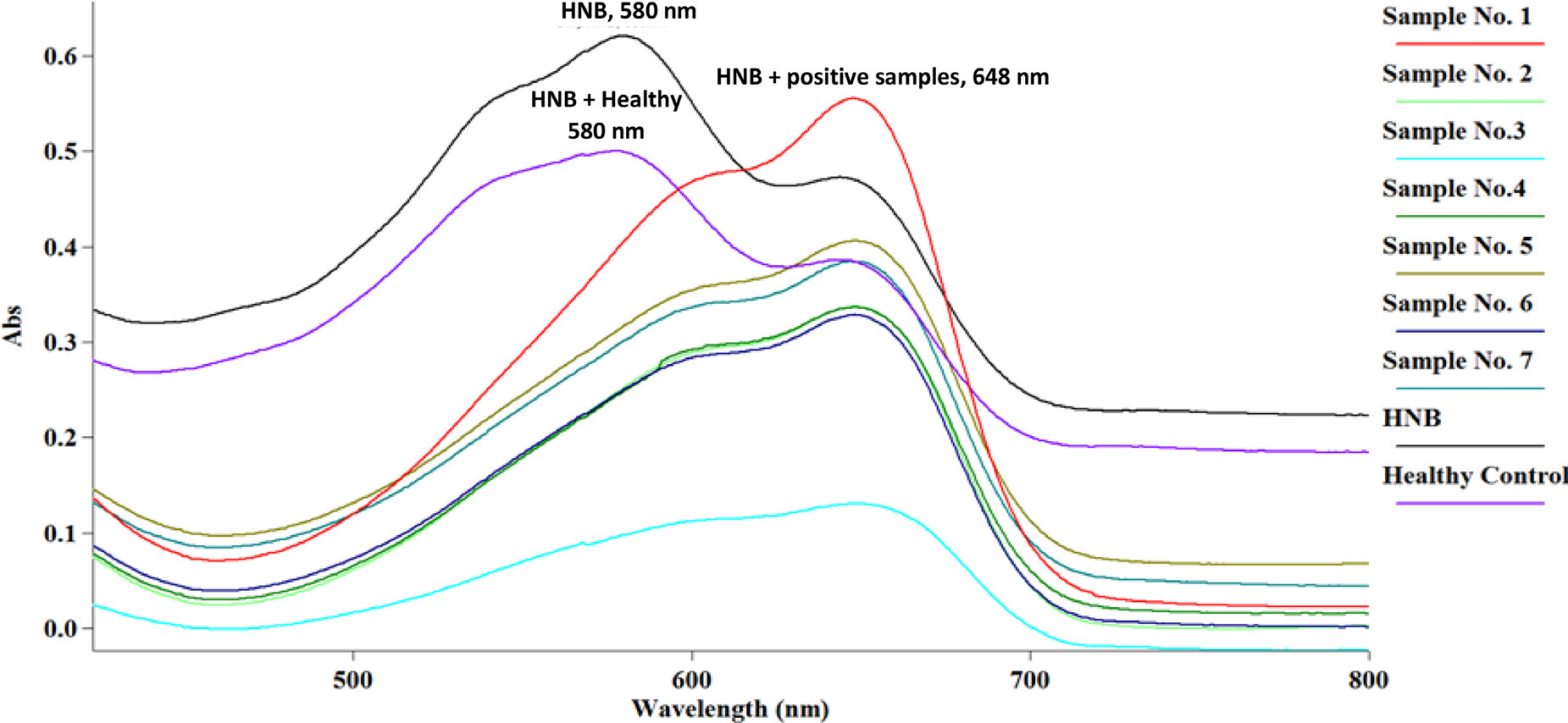
Absorbance of COVID positive samples with HNB and Healthy control.

LAMP amplified samples with HNB were analyzed in the spectrum in a spectrophotometer. However, only HNB, HNB with a healthy control spectrum, were found at 580 nm. HNB with Covid-19 positive samples were recorded at 648 nm, as shown in Fig. 7.

A total of 40 blood samples were processed out them; 33 samples were positive for LAMP and Q-PCR analysis, one sample showed positive in LAMP and negative in Q-PCR, 2 were negative in LAMP but positive in Q-PCR, and 4 blood samples were negative in both LAMP and Q-PCR. The amplified product was run on 2% agarose gel to identify the amplified product. The prevalence of SARS-CoV-2 disease was calculated as 87.5%. The positive and negative Likelihood ratios were found at 4.714 and 0.0715, respectively. The accuracy of LAMP was calculated as 92.50%, with a sensitivity of 94.28% and specificity of 80%. The positive predicted value for this test was 97.05%, while the negative predicted value was 66.67%.

### Cross-reactivity

The cross-reactivity of study specimens that were negative for Coivd-19 but positive for viruses like HBsAg, anti-HCV and HIV co-infected samples that could cross-react with Coivd-19 or co-infected viruses. No cross-reactivity was found among other viral RNA samples.

## Discussion

Ongoing pandemic situations of SARS-CoV-2 infection require extensive testing, preferably timely diagnosis in managing the disease. Therefore, this task evaluates the LAMP-based colorimetric method to rapidly detect SARS-CoV-2 in RNA extracted from a patient's blood to address this need. As the available method for detecting SARS-CoV-2 from NP and OP swab samples, it can give information on the presence of SARS-CoV-2 viral load majority by the quantification process in Q-PCR, NGS, CRISPR, dd-PCR etc. in hospitals. Collection of NP swabs are uncomfortable and must be carefully performed by a trained health worker using personal protective consumables. However, the technique have their own limitations in this pandemic situation. Despite the results from many other blood-based measurements, clinical chemistry and hematological parameters are also required to manage the severity in SARS-CoV-2 infected patients. Apart from those radiological investigations, contrast tomography chest score was helped in making of diagnosis. Though, 75% of the Q-PCR negative cases showed chest CT findings, with 48% likely to be positive for SARS-CoV-2 infection [11]. Thus, we have developed a simple, inexpensive and independent colorimetric protocol for detection of SARS-CoV-2 RNA.

All admitted patients were initially screened from the NP and OP swab samples based on their Q-PCR results. The blood samples were collected for routine investigations such as hematological, cytokines and clinical chemistry tests. The complete blood count viz. Hb, RBC, HCT, MCH, and MCHC values were deranged in the covid-19 positive patient compared to healthy subjects, while other studies have similar findings in Covid-19 infected patients [12], [13], [14]. In our study, there was an elevation in ALT, AST, WBC, ferritin, and inflammatory markers like CRP, LDH, and IL-6 levels in covid-19 patients, and similar findings were reported in other studies [15], [16], [17], [18]. The sensitivity and specificity of LAMP reaction with Q-PCR were 94.28% and 80%, respectively, with an accuracy of 92.50%.

Colorimetric LAMP on blood samples has broad potential to increase Covid-19 screening speed and capacity and also has high flexibility in implementation depending on equipment availability. Extension of the testing to asymptomatic individuals and increased test frequency could promote the application of predictive, preventive, and personalized medicine [19]. Expanding tests will improve the predictive reliability of modeling disease spread through better containment policies and identifying as well as protecting the vulnerable population [20]. Various LAMP assays have been developed and validated in swab and saliva samples of Covid-19 patients [21], very few have been commercialized due to cross-reactivity and lack of sensitivity in the assays [22].

LAMP method has been used to detect various viruses such as Herpes zoster [23], Dengue virus [24], Zika Virus [25], adenovirus [26], Hepatitis Virus [27], Cytomegalovirus [28], and Middle East Respiratory Syndrome (MERS-CoV) [29]. It can also be used for the detection of Human Fungal infection, *Pneumocystis pneumonia* [30]*,* human respiratory pathogens *Mycobacterium tuberculosis* [31,32] and Streptococcus pneumoniae [33], and protozoan infection malarial parasite (*Plasmodium falciparum*) [34]. LAMP can also detect aflatoxins by Aspergillus flavus [35].

We have developed the LAMP method in blood-based RNA isolation and validated the LAMP reaction process in Covid-19 positive samples with the Q-PCR and spectrophotometric evaluation at 648 nm. In this way, we have clearly shown that the negative for Covid-19 viral RNA, the absorbance was recorded at 580 nm after processing the samples in the LAMP method. The spectrum of LAMP reaction has clearly drawn the attention that the samples do not have covid viral RNA. Thus the result showed, LAMP assay is promising for diagnosis of Covid-19 viral RNA, epidemiological studies, and treatment monitoring of patients.

## Conclusion

LAMP is an emerging gene amplification technology. It is a quick and easy diagnostic tool for rapidly detecting and identifying viral infections. LAMP is easy to use, easily adaptable to all field conditions and environments, and has all the features you need for real-time assays, especially with high sensitivity. LAMP is also a convenient and valuable tool for developing countries as it is easy to use without needing advanced equipment or expert intervention. This ideal point-of-care testing opportunity meets the tremendous need for rapid and reliable on-site diagnostics urgently required in this pandemic. Thus, blood samples can be used for both various investigation as well as detection of SARS-CoV-2 RNA by LAMP method.

## Author contribution

SP conceived and designed the experiments. SP, Priyatma, PKP and RA contributed with experimental work and analysis tools. SP, Priyatma, PM and SA analyzed the data. SP wrote the paper in collaboration with all Co-Authors. All authors have read and approved the final manuscript.

## Funding agency

The research receives funding grants from Intramural grants, AIIMS, New Delhi.

## Declaration of Competing Interest

The authors declare no competing interests.

## Data Availability

Data will be made available on request.
